# A Computational Study on the Neck‐Stem Rectangular Tapered Connection: Effects of Angular Mismatch, Assembly, and Cyclic Loading

**DOI:** 10.1002/cnm.3909

**Published:** 2025-02-19

**Authors:** R. Cromi, L. Ciriello, F. Berti, L. La Barbera, T. Villa, G. Pennati

**Affiliations:** ^1^ Laboratory of Biological Structure Mechanics, Department of Chemistry, Materials and Chemical Engineering “Giulio Natta” (LaBS) Politecnico di Milano Milano Italy

**Keywords:** bi‐modular hip prosthesis, fatigue resistance, finite element analysis, modular neck design

## Abstract

The bi‐modular hip prosthesis is characterized by two tapered connections: a circular cross‐section at the head–neck interface and a rectangular cross‐section at the neck‐stem interface. Even if the latter guarantees customization, it concerns a high rate of early failure. The connection resistance is relatable to machining (tolerances cause angular mismatch), implantation (hammering force or manual), and usage (Body Mass Index [BMI]). Due to the lack of literature about the neck‐stem coupling, this work aims to investigate how the geometry of the rectangular taper connection and the external loads affect the fatigue strength of a bi‐modular hip prosthesis through a 3D Finite Element Model (FEM). Nine combinations of neck‐stem coupling are obtained considering the tolerances' limits on frontal and lateral angles as 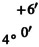. The CoCr neck and the Ti6Al4V stem, studied in their halved, are constrained and loaded inspired by the standard ISO 7206: the stem is distally encastered simulating the embedding and tilted by 10° concerning the sagittal plane, while the force is applied vertically. First, the influence of the assembly is investigated using 0.3kN, 2kN, and 4kN; then, a cyclical vertical force varying from 2.67kN to 5.34kN is imposed. Finally, one combination is analyzed in its integrity to evaluate the effect of the out‐of‐plane load. The study's findings concern: (i) a positive angular mismatch, which is responsible for proximal contacts, improves fatigue life, reducing *Sines* stress up to 33%; (ii) the higher the assembly force the higher the neck stability and the lower the extension of the overstressed lateral area; (iii) the implant fatigue resistance is directly proportional to the patient's BMI; and (iv) the out‐of‐plane external load causes a 40% increment in the fatigue failure risk.

## Graphical abstract

This work aims to investigate how the geometry of the neck‐stem rectangular taper connection, the assembly force, and the external load affect the fatigue strength of a bi‐modular hip prosthesis through 3D FEM. The study's findings concern: (i) a positive angular mismatch improves fatigue life; (ii) the higher the assembly force the higher the neck stability; (iii) the implant fatigue resistance is directly proportional to the patient's BMI; and (iv) the out‐of‐plane external load increases by 40% the fatigue failure risk.

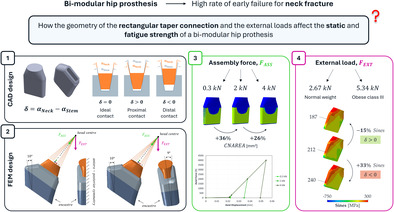

**Abbreviations**
δ
: angular mismatch
BMI: Body Mass Index
CoCr: chromium‐cobalt
*F*_ASS_
: assembly force
FEM: Finite Element Model
*F*_EXT_
: external load
Ti6Al4V: titanium

## 1 Introduction

**FIGURE 1:**
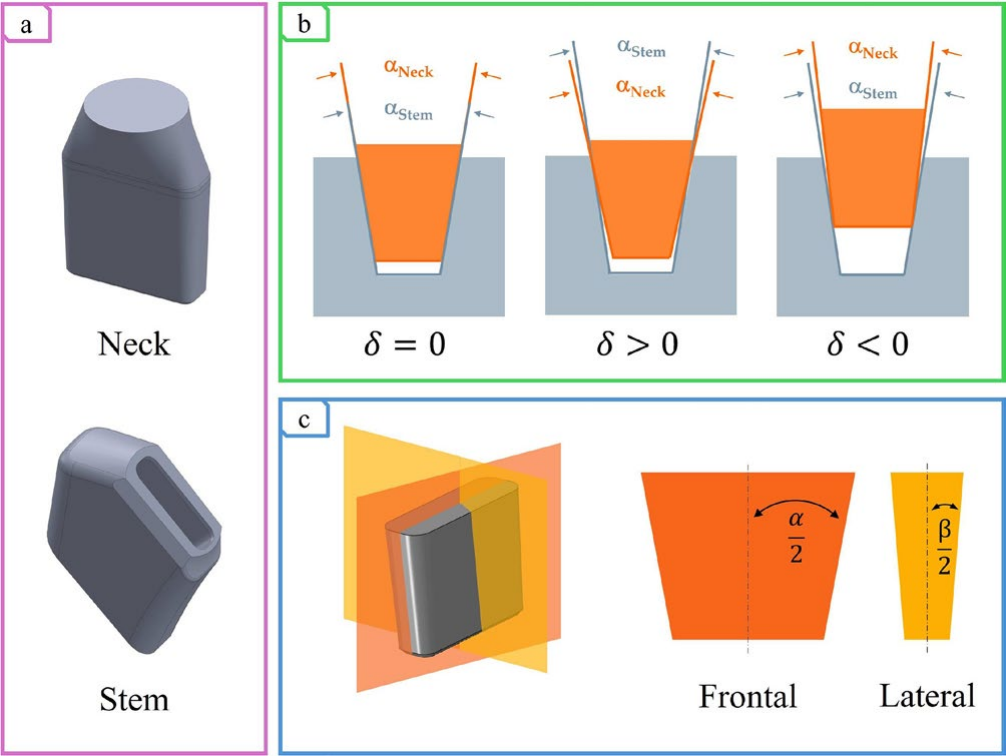
Rectangular taper connection: (a) 3D CAD geometry of the neck and stem; (b) Angular mismatch cases dependent on the definition as the difference between the neck and stem angles dimension; (c) Evidence of the frontal and lateral angles, α and β, which are responsible for the rise of different angular mismatch, $\delta_{\alpha }$ and $\delta_{\beta }$, respectively.

The hip joint is the spherical connection between the femur head and the acetabulum [1]; connecting the upper body with the limbs, it is responsible for load transmission and movement. When degenerative, inflammatory, congenital, or traumatic processes occur, it is necessary to deal with a prosthetic replacement [2]. To enable better biomechanical restoration and reduce revision surgeries, modularity has been introduced, allowing for customization with components of different materials, inclinations, and dimensions that can be coupled together [3]. Focusing on the stem component, modular and bi‐modular hip prosthesis can be distinguished. Both of them are characterized by a circular cross‐section *Morse* taper between the head and the neck, but the latter shows an additional rectangular cross‐section taper between the neck and the stem (Figure 1a). The taper connections are obtained thanks to manufacturing processes which are responsible for tolerances: the final dimensions commonly differs from the nominal ones, steaming dimensional mismatches between male and female. Specifically, the angular mismatch (δ) is defined as the difference between the neck and the stem angles (Figure 1b) [4].

Despite the theoretical advantages of modularity, as the patient‐specificity, and the common use of the head–neck connection, the neck‐stem one is still investigated due to a significant early failure rate (1.4% after 2 years [5]), attributable to corrosion, fretting, wear, and mechanical fatigue. In particular, corrosion and wear are associated with the opening of cracks medially and subsequently liquid entering, while fretting compromises the implant starting from the lateral surface [3]. Both corrosion and fretting are known to reduce the materials' fatigue life [6] and can't be considered separately: the micromotions disrupt the passive oxide layer exposing the material to corrosion which generates a crack able to propagate thanks to the two phenomena acting simultaneously. Corrosion is related to material coupling and the aggressive body environment while fretting is caused by micromotions related to the tilting phenomena: the neck, loaded with an external force, rotates in the stem housing [7, 8]. Given the presence of angular discrepancies, a positive δ seems to limit the micromotions at the interface [9]. Moreover, micromotions are inversely proportional to neck's material stiffness and assembly force ($F_{\mathrm{ASS}}$). In terms of material, if titanium (Ti6Al4V) is associated with less corrosion and ion release‐related problems, chromium‐cobalt (CoCr) is preferable for fretting and fatigue resistance due to its doubled *Young Modulus* [5, 10, 11]. On the other hand, although literature suggests a minimum requirement hammering of 4kN [12], ex vivo and in vitro experiments showed that surgeons apply a 2kN $F_{\mathrm{ASS}}$ on average [13]. To properly investigate the impact of multiple variables in terms of contact zones and pressures as well as localized stresses, the development of a Finite Element Model (FEM) seemes to be a good solution [11, 14]. To the best of the authors' knowledge, the FEM literature studies mainly refer to the circular head–neck connection [9, 12, 15, 16]. Such analyses revealed that circular *Morse* tapers' fatigue life can be improved by choosing a positive δ and applying a 4kN $F_{\mathrm{ASS}}$ [12]; conversely, young active male patients with a higher Body Mass Index (BMI) are commonly considered as risk factors for neck fracture in bi‐modular hip prosthesis but, even if the overstressed lateral surface seems to be responsible for crack nucleation in the majority of failure reports [5, 11, 17], its origination causes and avoiding strategies are still unclear. Moreover, differently from the classical *Morse* taper, which is considered symmetrical, the rectangular cross‐section implies a broader issue that includes tilting not only in the frontal plane but also in the transversal one [8], a dimensional mismatch [18] related to the depth and width of the tapered connection's section, and two different δ corresponding to α and β. Indeed, dealing with a *Morse* cone with a rectangular cross‐section no single angle can be defined; rather, two taper lines can be detected on its symmetry planes, as shown in Figure 1c.

Due to the above‐mentioned lack of literature, the purposes of this study are to (i) expand and clarify the concept and the effect of the angular mismatch, specifically related to a rectangular *Morse* taper and (ii) investigate the influence of $F_{\mathrm{ASS}}$ and external loads ($F_{\mathrm{EXT}}$) on the fatigue life of a bi‐modular hip prosthesis, focusing on the neck‐stem connection.

## 2 Methods

To take into account the influence of δ, $F_{\mathrm{ASS}}$, and $F_{\mathrm{EXT}}$, the workflow included the development of the neck and stem computer‐aided designs (CAD) followed by a FEM analysis to simulate the implantation and usage phases, depending on the surgeon and patient.

### 2.1 Geometry Effect

The neck and stem were constructed in *SolidWorks 2022* (*Dassault Systèmes; Vélizy‐Villacoublay, France*) as parametric 3D CAD (Figure 1a), depending on the frontal and lateral angles: α and β (Figure 1c). The definition of α and β as 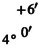 allowed obtaining four necks and four stems which, coupled together, resulted in nine different combinations (Table 1) where the nominal one, taken as a reference, was characterized by equal neck and stem angles and generated as a Boolean subtraction of the neck from the stem. The acceptable tolerance range was chosen considering that a δ equal to $6^{\prime }$ seemed to reduce the volumetric wear amount and improve the coupling stability [16]. Note that $\delta_{\alpha }$ can be detected medially and laterally, whereas $\delta_{\beta }$ can be detected anteriorly and posteriorly.

**TABLE 1:** Models generated from the frontal and lateral angles combinations, α and β, respectively: Mismatches $\delta_{\alpha }$ and $\delta_{\beta }$, derived by subtracting the stem angle from neck one, are also reported. On the right side, the run simulations are reported divided by the applied loads.

ID	$\alpha_{\text{Stem}}$	$\beta_{\text{Stem}}$	$\alpha_{\text{Neck}}$	$\beta_{\text{Neck}}$	$\delta_{\alpha }$	$\delta_{\beta }$	$F_{\mathrm{ASS}}$ $\left[\mathrm{kN}\right]$	$F_{\mathrm{EXT}}$	
								Normal	Obese III
Nominal	4°	4°	4°	4°	—	—	0.3	✓	✓
							2	✓	✓
							4	✓	✓
A	4°	4°	4°6′	4°6′	+6′	+6′	0.3		
							2	✓	✓
							4		✓
B	4°6′	4°6′	4°	4°	−6′	−6′	0.3		
							2	✓	✓
							4		✓
C	4°	4°6′	4°6′	4°	+6′	−6′	0.3	✓	✓
							2	✓	✓
							4	✓	✓
D	4°6′	4°	4°	4°6′	−6′	+6′	0.3		
							2	✓	✓
							4		
E	4°	4°	4°	4°6′	—	+6′	0.3		
							2	✓	✓
							4		
F	4°	4°6′	4°	4°	—	−6′	0.3		
							2	✓	✓
							4		
G	4°	4°	4°6′	4°	+6′	—	0.3		
							2	✓	✓
							4		
H	4°6′	4°	4°	4°	−6′	—	0.3		
							2	✓	✓
							4		✓

**FIGURE 2:**
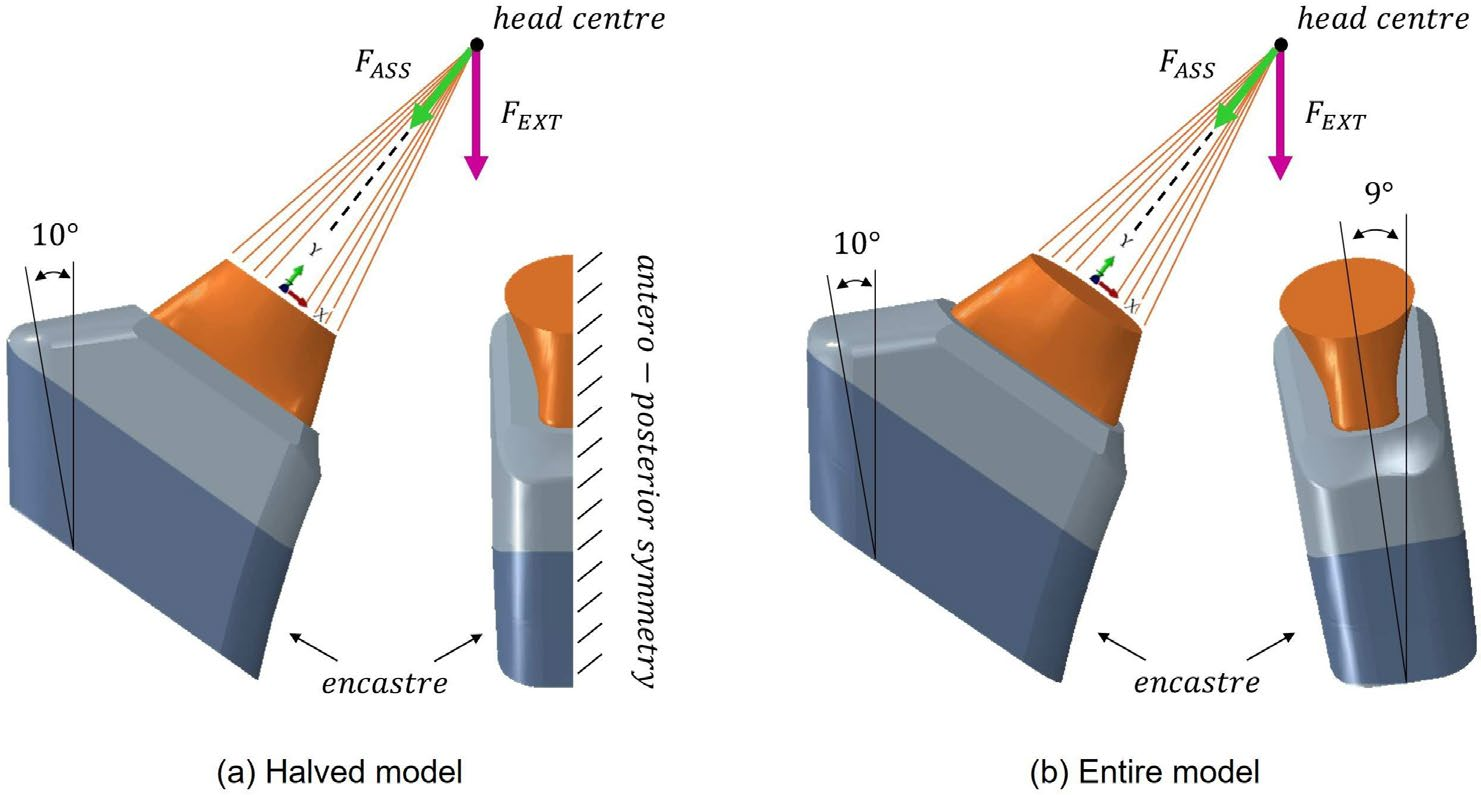
Model of the assembled prosthesis, in evidence the boundary conditions (antero‐posterior symmetry for the halved model (a) while 9° rotation with respect to the antero‐posterior plane for the entire model (b), stem's encastre and 10° rotation with respect to the sagittal plane), the numerical connection between the center of the head and the neck's upper surface, and the loads: coaxial assembly force ($F_{\mathrm{ASS}}$) and vertical external load ($F_{\mathrm{EXT}}$ ). Anterior and lateral views are reported on the left and right sides, respectively for each model.

Each CAD combination was imported in *ABAQUS/CAE 2022* (*Dassault Systèmes SIMULIA Corporation, Johnston, RI, USA*), where the models and the simulations were developed. Each 3D geometry was discretized using a hybrid meshing strategy: linear hexahedral elements (C3D8) were used within partitioned regions where contact occurred between the neck and the stem; linear tetrahedral elements (C3D4) were imposed everywhere else. A mesh sensitivity analysis was performed, with particular care to the areas of interaction between the neck and stem: the monitored quantities of interest were stiffness, stress components and *Von Mises* stress. The mesh strategy and the elements' dimensions were chosen as a compromise between the solution convergence in the contact zones and the computational time required. Specifically, the element's size was equal to 140μm and 185μm for the neck and the stem contact region, respectively. Elastic isotropic materials were assigned both to the CoCr neck (*E* = 230 GPa; ν=0.3 [19]) and the Ti6Al4V stem (*E* = 110 GPa; ν=0.3 [20]). The components were assembled by imposing an axial translation of the neck until at least one point of its taper surface reached the stem one. A *surface‐to‐surface* contact was set between the neck and stem with *hard* normal behavior and a friction coefficient equal to 0.3 defining the tangential behavior [21]. Due to the high computational time required and considering that the longitudinal component of the external load is typically the 85% of the total, simulations were based on half an implant, thus simplifying the ISO 7206 standard [22]. The assembly was rotated only by 10° with respect to the sagittal plane while an antero‐posterior symmetry was imposed instead of rotating by 9° with respect to the frontal plane (Figure 2a). Additionally, (i) the stem was encastred up to 10 cm from the top surface, mimicking the embedding level [22]; (ii) the neck and stem were simplified in their upper and lower part leaving only the rectangular *Morse* taper connection between the components to reduce the geometry variability and the computational time. The bi‐modular hip prosthesis was simulated in its main stages which are the assembly, by the surgeon, and the external loading once the device is implanted. Both the $F_{\mathrm{ASS}}$ (aligned with the neck axis and taper coupling, as identified with the *Y_axis* of a local reference system) and the $F_{\mathrm{EXT}}$ (vertical) were applied on subsequent simulation steps on the same reference point, positioned in the center of the head and connected to the upper face of the hip‐neck through a numerical rigid connection (Figure 2).

To verify the correctness of CAD design and FEM mesh processes, a check on the structures' sizes was performed both in *SolidWorks* and *ABAQUS/CAE*, ensuring that the nominal model is characterized by equal neck and stem dimensions.

### 2.2 Assembly Force Effect

To first evaluate the effect of δ with the same $F_{\mathrm{ASS}}$, and then that of a different $F_{\mathrm{ASS}}$ with the same δ, all models were subjected to three levels of load. A ramp force with different entities was simulated: 0.3kN, 2kN, and 4kN corresponding to manual insertion, as measured in our laboratory via preliminary experimental tests, average [13] and minimum required hammering [12], respectively. The contact area evaluation was performed using the *CSTATUS* and *CNAREA* variables in *ABAQUS/CAE* after an unloading phase that follows the application of $F_{\mathrm{ASS}}$. The *CNAREA* variable corresponds to the extension, in mm^2^, of the contact area, while the *CSTATUS* one shows the zones of interaction indicating in green the *sticking*, in light blue the *slipping* and in blue the *absence* of contact between the two surfaces. In addition, the displacement of the neck was studied to assess the position reached in the stem housing during the assembly phase and after the unloading; according to this, even the elastic deformation accounted by the neck could be estimated.

### 2.3 External Load Effect

**FIGURE 3:**
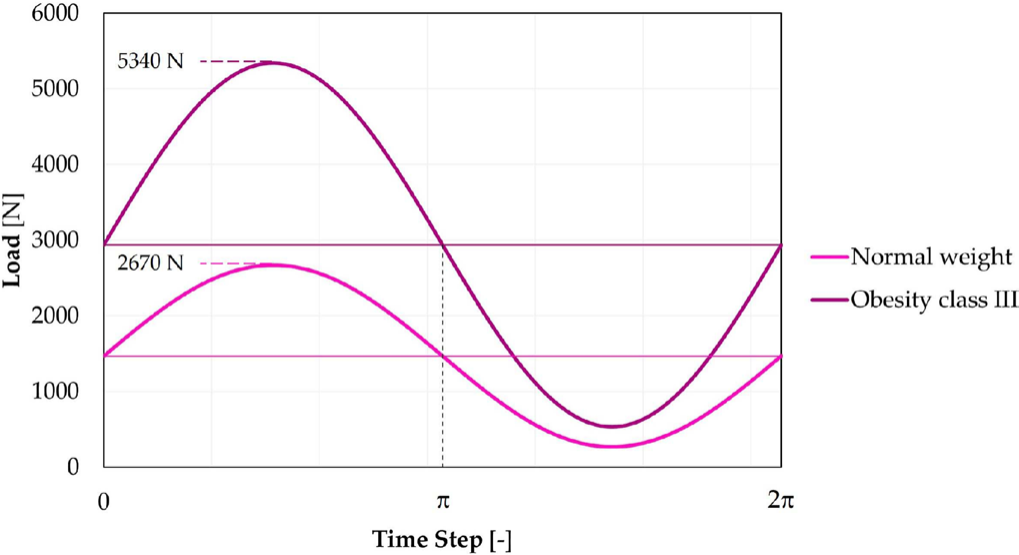
Trend of $F_{\mathrm{EXT}}$, characterized by R=0.1 and a maximum value of 2.67kN and 5.34kN, representative of the implant usage by a normal weight and class III obesity patient, respectively.

Following assembly and unloading, a vertical force was applied at the head center for five load‐unload cycles (Figure 2). $F_{\mathrm{EXT}}$ is characterized by R=0.1 and two different maximal entities, 2.67kN and 5.34kN (Figure 3); starting from the last value, which depends on the ISO 7206 standard [22], the other one was based on its 50%. Considering the average Italian population height added to the quadruplicated weight loading on the hip during the monopodalic support, they were associated with different BMI: class III obesity and normal weight, respectively.

The *Sines* multi‐axial fatigue criterion was used to evaluate the prosthesis resistance under the cyclic load

(1) $$\sigma_{\text{Sines}}=\sigma_{\mathrm{VM},a}+k\cdot I_{1,m}\leq \sigma_{\mathrm{FA}}$$

where $\sigma_{\mathrm{VM},a}$ is the triaxial alternate *Von Mises* stress; k, chosen as 0.35 [19], is the ratio between the bending fatigue strength limit, $\sigma_{\mathrm{FAf}}$, and the ultimate strength $\sigma_{R}$; $I_{1,m}$ is the mean first invariant. Considering $\sigma_{R}=1277\;\mathrm{MPa}$, the *Sines* stress limit, $\sigma_{\mathrm{FA}}$, indicating CoCr fatigue resistance, is calculated equal to 447MPa.

### 2.4 Out‐of‐Plane Load Effect

Knowing the ideality resulting from the standard simplification, as a preliminary analysis model *A* (Table 1) was investigated in its entirety so that the effect of the out‐of‐plane load (correspondent about to the 15% of the total ***F***_EXT_) on its fatigue life could be evaluated. In this case, the simulations were performed completely following the standard [22] so that the implant was rotated by 9° with respect to the frontal plane too, while $F_{\mathrm{EXT}}$ was maintained vertical (Figure 2b). The entire model was assembled with $F_{\mathrm{ASS}}=2\;\mathrm{kN}$ and then, after the unloading phase, five load‐unload cycles corresponding to normal weight and class III obesity patients were applied.

The choice of *A* as the model to be investigated in its entirety after the fatigue analysis on the halved models is reported in Subsection 3.2.4.

## 3 Results

### 3.1 Influence of Geometry and Assembly Force During the Coupling

Increasing $F_{\mathrm{ASS}}$ from 0.3kN to 2kN and then from 2kN to 4kN, there was an average increment in axial displacement of 36% and 26%, respectively (Table 2). Moreover, despite the application of an axial $F_{\mathrm{ASS}}$, a non‐null transversal displacement was detected: it was directly proportional to the $F_{\mathrm{ASS}}$ and assumed a maximal value of 0.53μm for the nominal model.

**TABLE 2:** Neck's axial displacement at the assembly ($F_{\mathrm{ASS}}$ equal to 0.3kN, 2kN, and 4kN) on the left column and axial displacement difference, called Δ, between the assembly pick and unload, due to the elastic return, on the right column. The related *Force—Axial Displacement* graphs can be visualized in the Data S1: Figures A–C.

ID	$F_{\mathrm{ASS}}$					
	0.3kN		2kN		4kN	
	Load [μm]	Δ [μm]	Load [μm]	Δ [μm]	Load [μm]	Δ [μm]
Nominal	23.3	1.1	37.6	3	54.5	5.2
A	61.1	1.4	177	3	243	6
B	28.6	1.4	81.5	4.2	119	7
C	27.2	1.5	91.7	4.5	136	8
D	99.2	2.1	258	6	363	9
E	16.4	1.2	78.3	3.4	138	6
F	27.7	1.5	91.4	4.5	134	7
G	23.6	1.1	45.7	3.2	69.8	5.5
H	90.5	2.1	241	5	335	9

**FIGURE 4:**
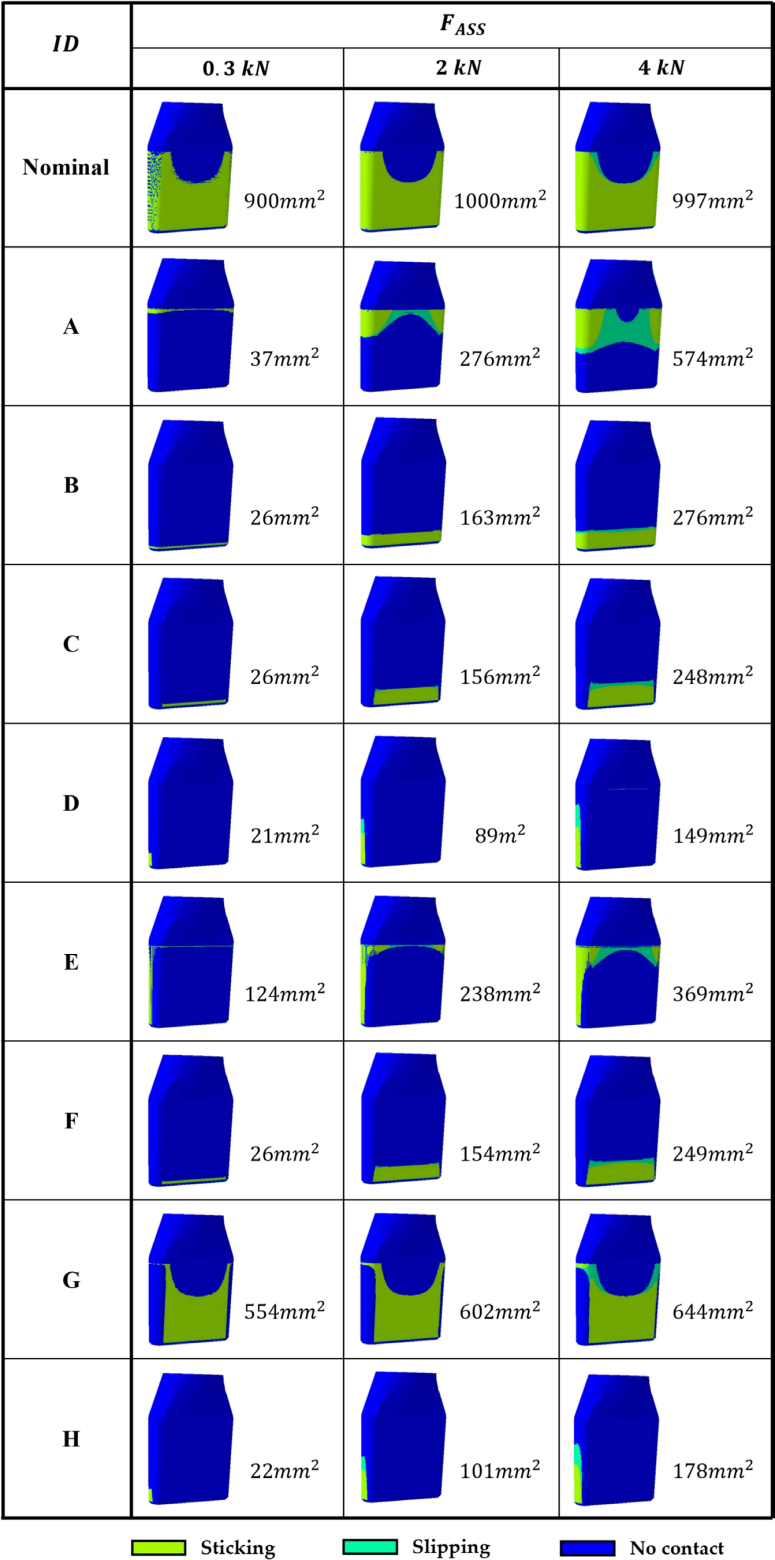
*CSTATUS* color map and *CNAREA* value in mm^2^ for all models after the assembly and the unloading phases. Specifically, the neck's insertion is simulated as it is performed manually or by applying an average and a minimum required hammering, equal to 0.3kN, 2kN and 4kN, respectively.

The *CSTATUS* variable showed that homogeneous (Figure 4, Nominal model), proximal (Figure 4, Model *A*), and distal (Figure 4, Model *B*) contacts corresponded to null, positive, and negative δ, respectively. The nominal model exhibited the absence of interaction on the frontal‐proximal tapered surface. Moreover, as it was possible to observe in Figure 4, a negative δ, causing a distal interaction, did not allow proximal and homogeneous contacts to occur as shown by the models *C*, *D*, *F*, and *H*.

### 3.2 Influence of Geometry, Assembly Force and External Loads During the Dynamic Loading

#### 3.2.1 Geometry Effect

Applying to all models $F_{\mathrm{ASS}}=2\;\mathrm{kN}$ and a normal weight cyclic $F_{\mathrm{EXT}}$, it was possible to compare the influence of δ on the implant fatigue resistance. All models did not exceed the limit value of *Sines* stress. However, the maximum *Sines* on the lateral traction surface showed a dependence on the angle discrepancy: considering a normal weight $F_{\mathrm{EXT}}$ a negative or positive δ implied an increment of 33% (Table 1, Model *B*) and a reduction of 15% (Table 1, Model *A*), respectively, compared to the nominal case (Table 1, *Nominal* Model). Moreover, $\delta_{\alpha }$ had a grater influence on the prosthesis' fatigue life compared to $\delta_{\beta }$: the decrease in *Sines* was around 11% (Table 1, Model *G*) and 3% (Table 1, Model *E*) if only $\delta_{\alpha }$ and $\delta_{\beta }$ positive were considered, respectively. Similarly, increases of 27% (Table 1, Model *H*) and 4% (Table 1, Model *F*) were observed at negative $\delta_{\alpha }$ and $\delta_{\beta }$, respectively. For further information, please refer to the Data S1: Figure D.

#### 3.2.2 Assembly Force Effect

Analyzing the nominal model, at the same δ and BMI, the maximum value of the *Sines* stress was independent of $F_{\mathrm{ASS}}$. However, if $F_{\mathrm{ASS}}$ increased, the lateral overstressed zone's extension reduced progressively.

**FIGURE 5:**
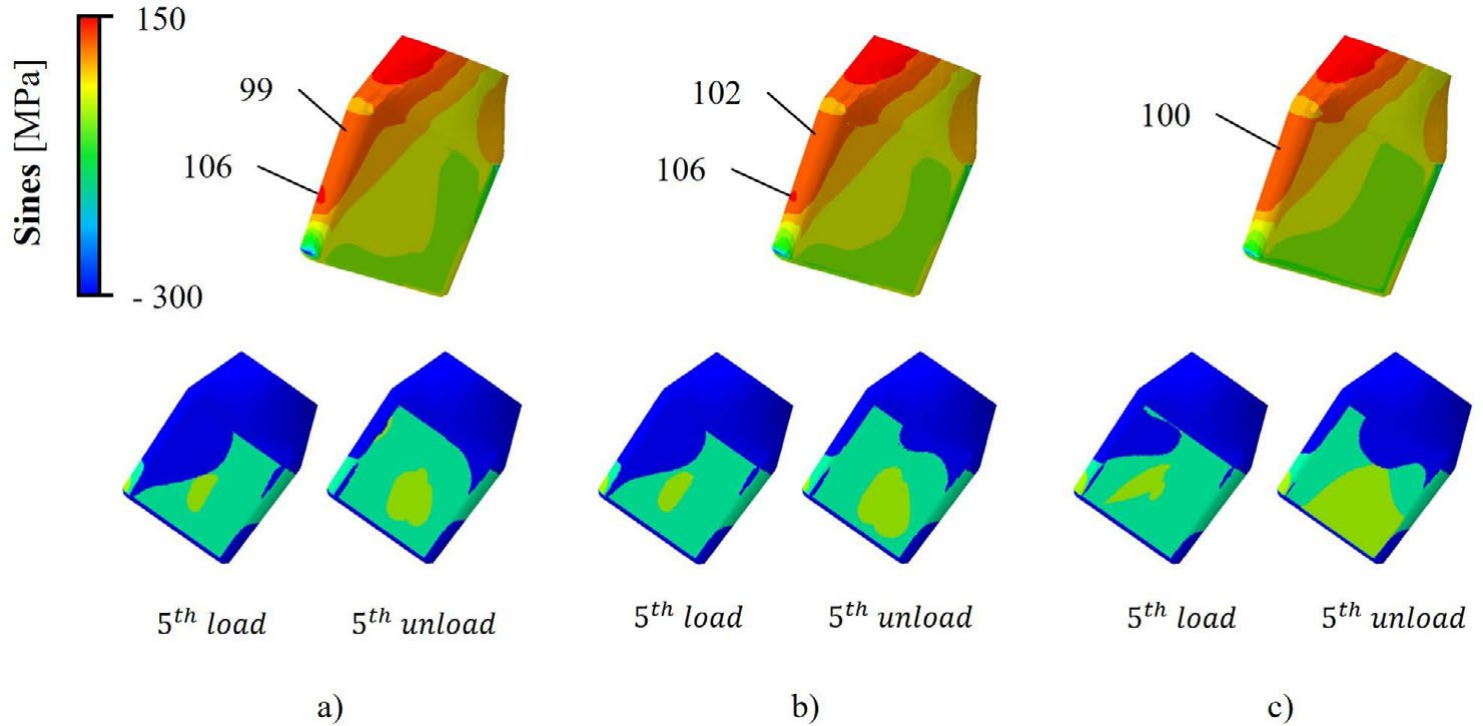
*CSTATUS* and *Sines* stress color map of the nominal model at the 5th load‐unload cycle. Evidence of the maximum values related to normal weight $F_{\mathrm{EXT}}$ and different $F_{\mathrm{ASS}}$ entities: (a) 0.3kN; (b) 2kN; (c) 4kN.

A neck's tilting in the frontal plane was detected. The neck rotated around a central sticking zone causing a change in contact zones which depended on the $F_{\mathrm{ASS}}$: from 0.3kN to 2kN and then from 2kN to 4kN the adhesive area increased by 17.2% and 28.6%, respectively for the nominal case (Figure 5).

#### 3.2.3 External Loads Effect

**FIGURE 6:**
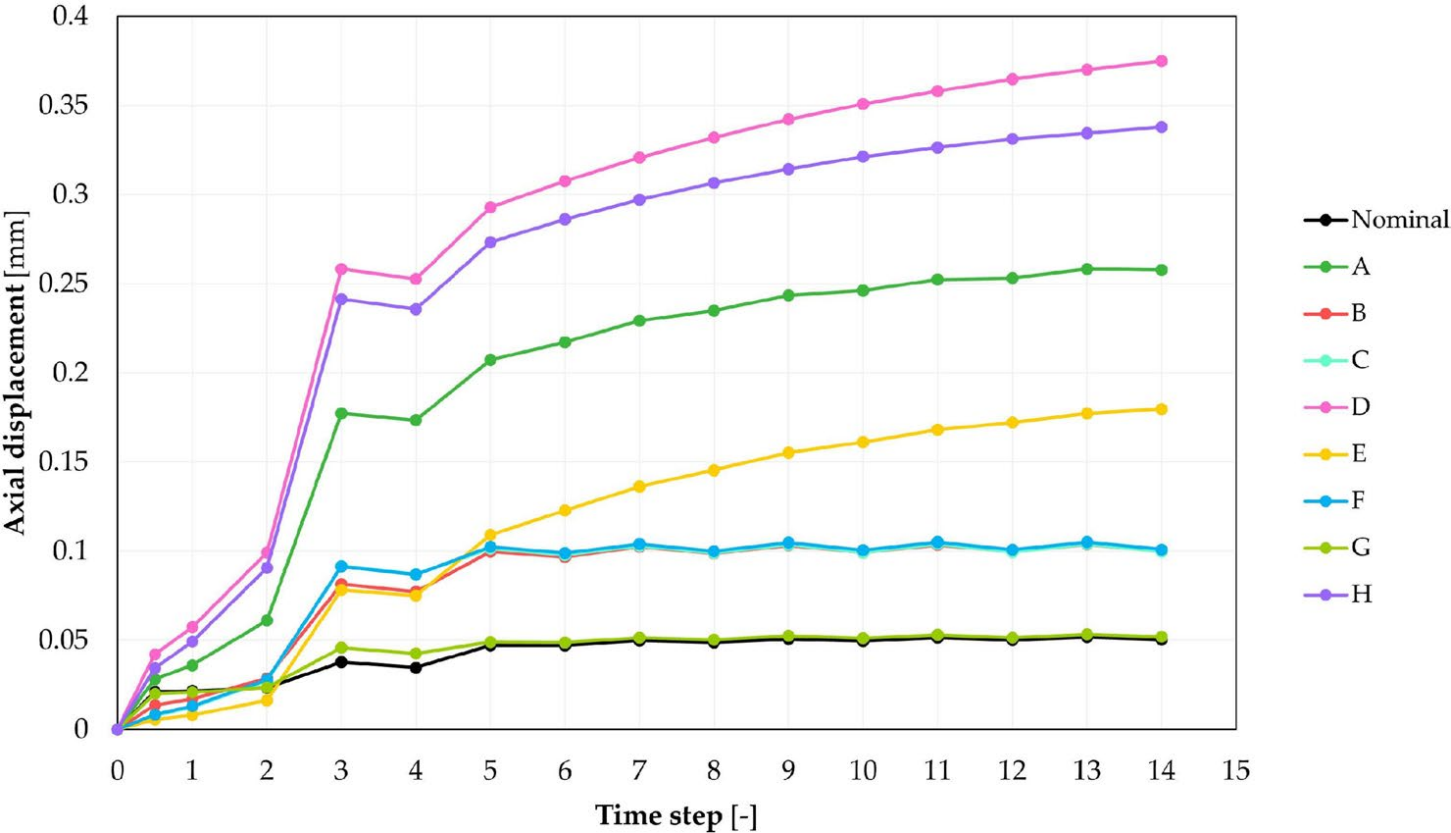
Axial displacement—Time steps graphs for all models assembled with $F_{\mathrm{ASS}}=2\;\mathrm{kN}$ and then cyclical loaded with $F_{\mathrm{EXT}}=5.34\;\mathrm{kN}$, corresponding to a class III obesity patient.

Observing the graph in Figure 6, it could be noticed that when a 2kN $F_{\mathrm{ASS}}$ and a class III obesity $F_{\mathrm{EXT}}$ were applied, not all combinations reached stability in five cycles: some of the analyzed combinations showed an always increasing neck's axial displacement, which did not reach any *plateau*, indicating a continuous translation without steadying at a constant position.

Focusing on the *CSTATUS* variable (Data S1: Figure D), it could be noticed that the tilting phenomenon depended on the BMI: the higher the $F_{\mathrm{EXT}}$ the lower the sticking area extension and the enhanced the slipping one.

Considering the nominal model, if δ and $F_{\mathrm{ASS}}$ were maintained constant, doubling the BMI from a normal weight to an obese class III condition, the increased cyclic load caused a doubled *Sines* maximum lateral stress that passed from 106MPa to 212MPa. Keeping constant $F_{\mathrm{ASS}}$, a class III obesity load caused an increment of *Sines* stress of about 40% on average for all geometry combinations.

#### 3.2.4 Out‐Of‐Plane Load Effect

**FIGURE 7:**
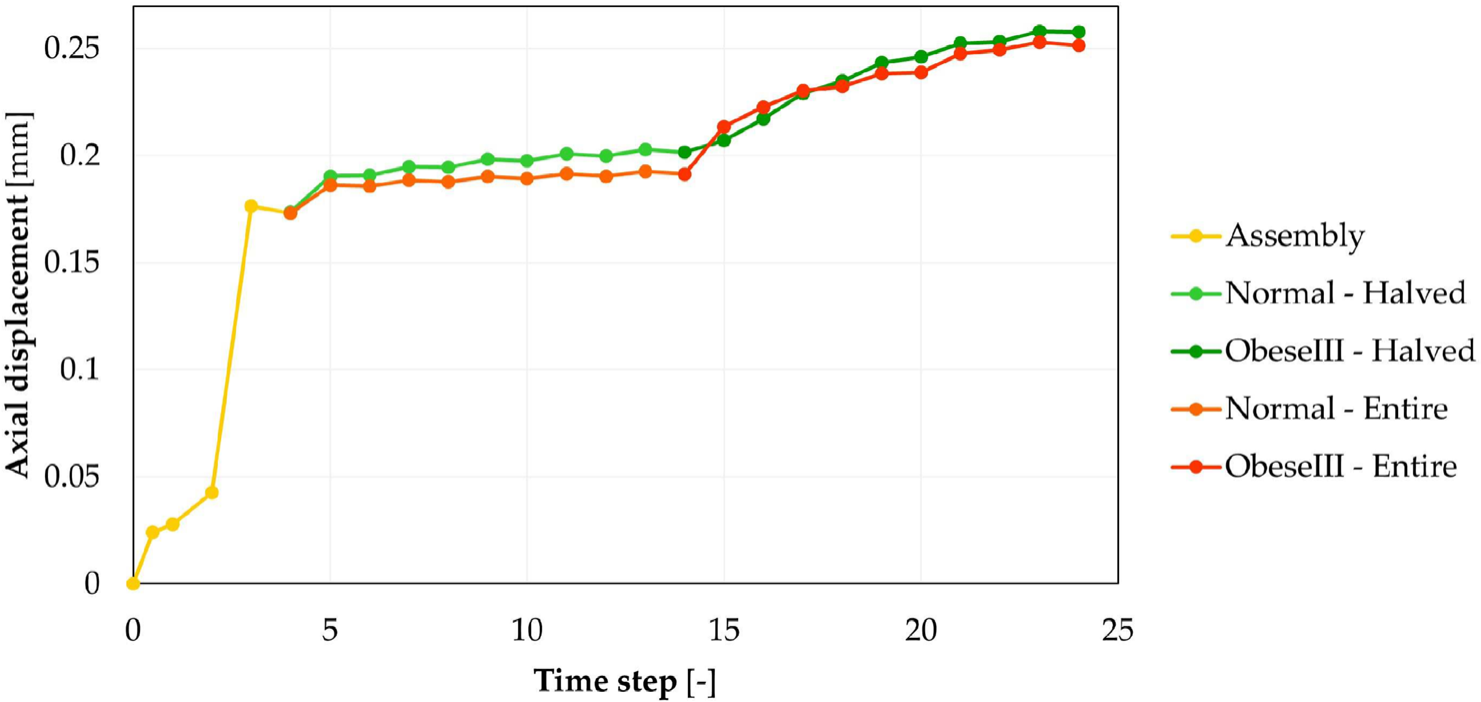
Axial displacement—Time steps graphs for the model *A* assembled with $F_{\mathrm{ASS}}=2\;\mathrm{kN}$ and then cyclical loaded with $F_{\mathrm{EXT}}$, corresponding to a normal weight and a class III obesity patient, respectively. The comparison between the halved and the entire model is reported.

Model *A* was chosen as the combination to be studied in its entirety downstream the *CSTATUS* analysis in the poximo‐lateral contact zones after a 2kN assembly and $F_{\mathrm{EXT}}$ associated with a class III obese patient were applied. Comparing the entire model *A* with the halved one, a 5% and a 2% neck displacement reduction was seen during the normal weight and the class III obesity cyclic $F_{\mathrm{EXT}}$, respectively (Figure 7).

**FIGURE 8:**
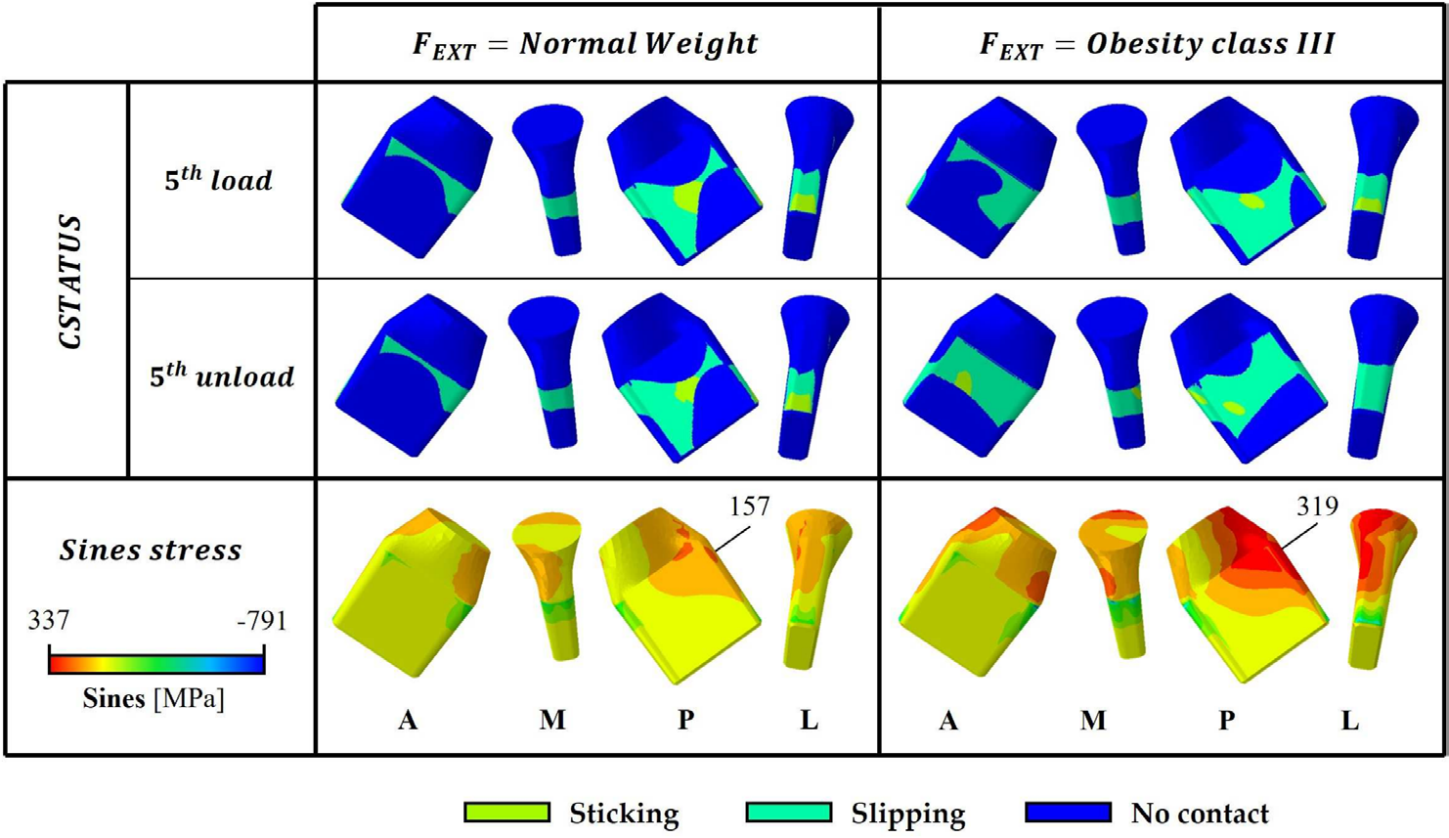
*CSTATUS* and *Sines* stress color map at the 5th load‐unload cycle, where lateral maximum values are highlighted. The model *A*, simulated in its integrity, is assembled with $F_{\mathrm{ASS}}=2\;\mathrm{kN}$ and then loaded with a normal weight and a class III obesity $F_{\mathrm{EXT}}$, respectively on the left and on the right side. The anterior (A), medial (M), posterior (P), and lateral (L) views are reported.

The out‐of‐plane $F_{\mathrm{EXT}}$ was responsible for the neck's tilting in the transversal plane as well as in the frontal one: on the postero‐lateral neck area a slipping zone was detected and no more sticking was present. Even in this case, the augmentation in the BMI was responsible for a linearly proportional increase in the maximum lateral *Sines* stress. Examining the influence of the out‐of‐plane cyclic load there was an increase in the highest fatigue stress greater than 41%; even though, 319MPa was a lower value than the limit one. Moreover, the overloaded lateral area remained proximal, but it underwent a shift to the transition between the lateral and posterior surfaces (Figure 8).

Considering the time required to simulate the implant surgical assembly and patient usage, the entire model, compared to the halved one, needed an increment in the computational time demand (185 h vs. 22 h).

## 4 Discussion

Bi‐modular hip prosthesis early failure rate does not allow the full exploitation of all modularity benefits such as the implant customization based on the patient peculiarities. Many researches clearly state that these implants must be avoided in young obese patient [3, 5, 23]. However, to the best of the authors' knowledge, there is a lack of literature regarding all the potential features and causes implied in the neck‐stem tapered junction malfunction. This work aims to systematically analyze the effect of angular discrepancy, surgical assembly, and external loads on the bi‐modular hip prosthesis fatigue resistance through FEM.

Analyzing the *CSTATUS* variable after the assembly and unloading phases, it can be noticed that the nominal model shows the absence of contact on frontal‐proximal tapered surface (Figure 4, *Nominal* Model) even if a uniform interaction was expected. The dimensional check done both on the *CAD* and *FEM* models suggests that probably when the neck is made to enter the stem housing, a Poisson's effect occurs and the different deformation of the two structures generates a gap. The lack of contact showed by the models *C*, *D*, *F*, and *H* (Figure 4) can be explained by considering that a previous distal contact does not allow proximal or homogeneous ones to be exploited on the taper's surfaces because the first distal interaction creates a shape constraint that stops the neck's axial displacement.

Despite a coaxial $F_{\mathrm{ASS}}$ is applied, a non‐null transversal displacement is present; this phenomenon indicating the neck's auto‐centering is caused by the different lateral and medial stem stiffness providing an unsymmetrical constraint for stem's axial advancement during assembly.

The neck's advancement is directly proportional to the $F_{\mathrm{ASS}}$ and inversely proportional to the *CNAREA*: a lower contact area (Figure 4) is responsible for local overloading that causes greater deformations and, consequently, a higher neck's shift (Table 2).

The neck's tilting in the stem housing on the frontal plane is caused by the non‐coaxial $F_{\mathrm{EXT}}$. This confirms the micromotions theory presented by Falkenberg et al. [7]: the neck switches from a mirrored interaction between the medial and lateral surfaces detected after the assembly phase, to an unsymmetrical one when the usage is simulated (Figures 4, 5 and D‐Data S1). However, (i) during the load the contact is localized proximally and distally on the medial and lateral sides, respectively, without a complete interaction on the medial surface; (ii) during the unload the contact remains qualitatively almost the same, with a small increment in the interaction area extension.

The *Sines* stress discrepancy dependent on δ confirms what have been stated in the literature [9, 15, 16] about the ability of a positive δ to improve fatigue life; larger δ seems to be preferable because positive δ combinations exhibit a lower *Sines* stress than the nominal case, where the angle discrepancy is null, although this does not find all the analyzed literary studies in agreement. Nevertheless, since the neck taper it's characterized by a rectangular cross‐section it must be considered the greater influence of $\delta_{\alpha }$ on the prosthesis' fatigue life compared to $\delta_{\beta }$, which may be justified considering that the first one is responsible for medio‐lateral contacts that enable the neck's tilting and bending reductions.

The choice of the entire model *A* to be analyzed according to the standard [22] depended on the interaction at the proximo‐lateral taper surfaces, considered the area from which cracks nucleate; specifically, a transition between sticking and slipping, responsible for the highest risk of failure for the fretting fatigue phenomena [24], was detected. Applying the $F_{\mathrm{ASS}}=2\;\mathrm{kN}$ and $F_{\mathrm{EXT}}=5.34\;\mathrm{kN}$, as in the halved model, no more sticking can be noticed at the proximo‐lateral zone (Figure 8); however, this can be justified considering that the entire model did not reach a stable position when five load‐unload cycles are applied. The entire model's lower final axial displacement compared to the halved one is due to a smaller axial component of the $F_{\mathrm{EXT}}$ caused by the implant 9° rotation with respect to the frontal plane.

The entire model growth in the maximum *Sines* stress value with respect to the same combination analyzed considering an antero‐posterior symmetry can be attributable to the added neck's flexion caused by the out‐of‐plane external load. Assuming that a similar increment in the *Sines* stress occurs for all combinations when analyzed in their integrity, the model characterized by a homogeneous negative δ would reach a maximum value of 403MPa, which is really near to the limit one. Given the presence of slipping contacts around the overloaded area, wear and fretting phenomena can be hypothesized; knowing the significant reduction in metal fatigue life from fretting and corrosion, always present due to the aggressive body environment, it is conceivable that some models might fail under an external cyclic load. However, the bi‐modular hip prosthesis tapered neck seems to resist to a pure fatigue phenomena even when risk factors are simulated, as a class III obese patient [3, 23, 25].

## 5 Conclusions

This study confirmed the importance of considering geometry, $F_{\mathrm{ASS}}$ and $F_{\mathrm{EXT}}$ on the implant's expected fatigue life. A positive δ improves fatigue life by reducing till the 33% the maximum *Sines* stress in the traction area compared with the nominal. Specifically, the effect of $\delta_{\alpha }$ is almost four times higher than the effect of $\delta_{\beta }$. Considering the assembly phase, for a given δ, $F_{\mathrm{ASS}}$, or $F_{\mathrm{EXT}}$ causes greater advancement and increased interference in the taper connection, improving stability. On the other hand, for a given BMI, the maximum *Sines* stress seems to be independent of $F_{\mathrm{ASS}}$. However, the overstressed lateral area decreases in its extension as the $F_{\mathrm{ASS}}$ increases. Maintaining constant δ and $F_{\mathrm{ASS}}$, the BMI has two implications: (i) if the coaxial component of the $F_{\mathrm{EXT}}$ exceeds the $F_{\mathrm{ASS}}$ magnitude, an advancement of the neck occurs, increasing the interference between components; (ii) independently from the $F_{\mathrm{ASS}}$ , the *Sines* stress values intensify proportionally with the increasing in cyclic load, depending on the patient's BMI. The maximum registered *Sines* stress, being lower than the CoCr limit value, states that a pure fatigue phenomenon does not cause the bi‐modular hip prosthesis neck's fracture.

The work addresses some limitations related to the geometry and the boundary conditions that can be turned into future developments. The analysis focused on homogeneous taper variation; however, it would be interesting to consider the α/2 and β/2 semi‐angles separately (Figure 1c). Angular tolerances depend on manufacturing processes and are therefore randomly distributed; moreover, this scenario allows the exploitation of more realistic combinations concerning the milling cutter obtaining process. Moreover, both CoCr and Ti were defined as elastic material; in the perspective of a high‐fidelity model, the study and implementation of their plasticity behavior will be required. According to this, a more accurate description of the localized stress in the contact zone would be exploited. The model usability is confined because of the high computational time required even for the most simplified cases. All the more reasons, simulations on the entire implant were limited (computational time over 185 h); however, given the increase in *Sines* stress and antero‐posterior tilting, it would be interesting to expand the case study, especially for combination with negative δ, which are riskier in fatigue.

## Ethics Statement

The authors have nothing to report.

## Conflicts of Interest

The authors declare no conflicts of interest.

## Supporting information


Data S1.

## Data Availability

The data that support the findings of this study are available from the corresponding author upon reasonable request.
