# Supplementary material for: A Computational Study on the Neck‐Stem Rectangular Tapered Connection: Effects of Angular Mismatch, Assembly, and Cyclic Loading
Source: Int J Numer Method Biomed Eng. 2025 Feb 19;41(2):e3909. doi: 10.1002/cnm.3909 (PMC11837768; doi:10.1002/cnm.3909)
Supplement: Supplementary file 1 — Data S1. [file CNM-41-e3909-s001.pdf]

Supplementary Material

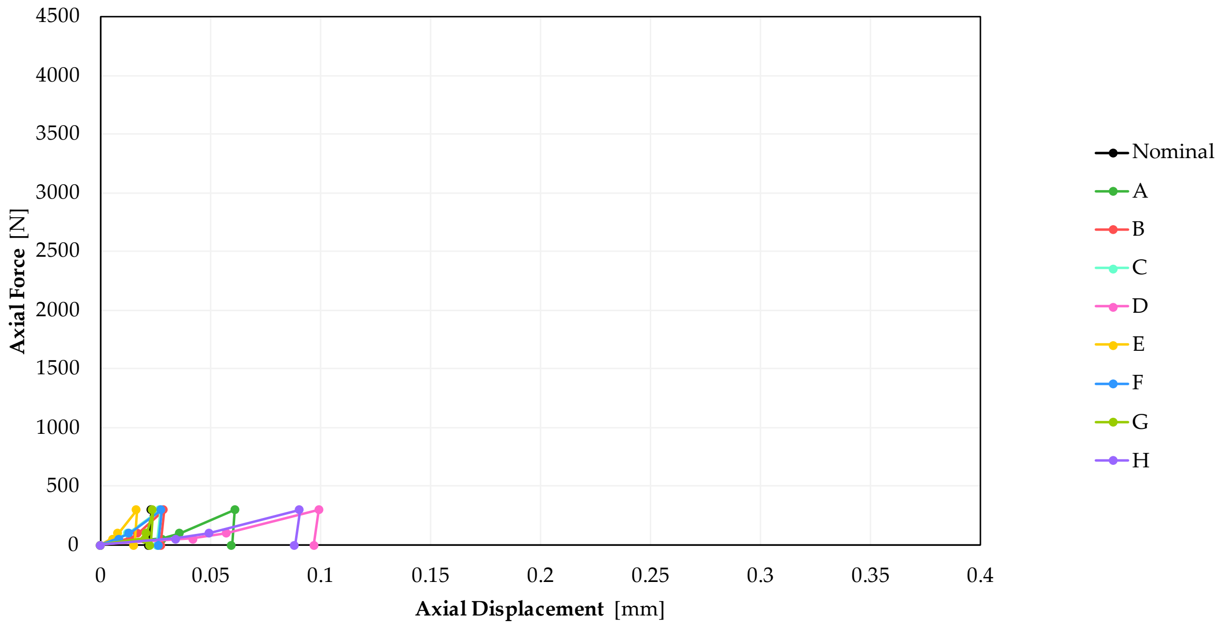

Figure A: Axial Force - Axial Displacement graphs for all models assembled with $F_{ASS} = 0.3kN$ and then unloaded.

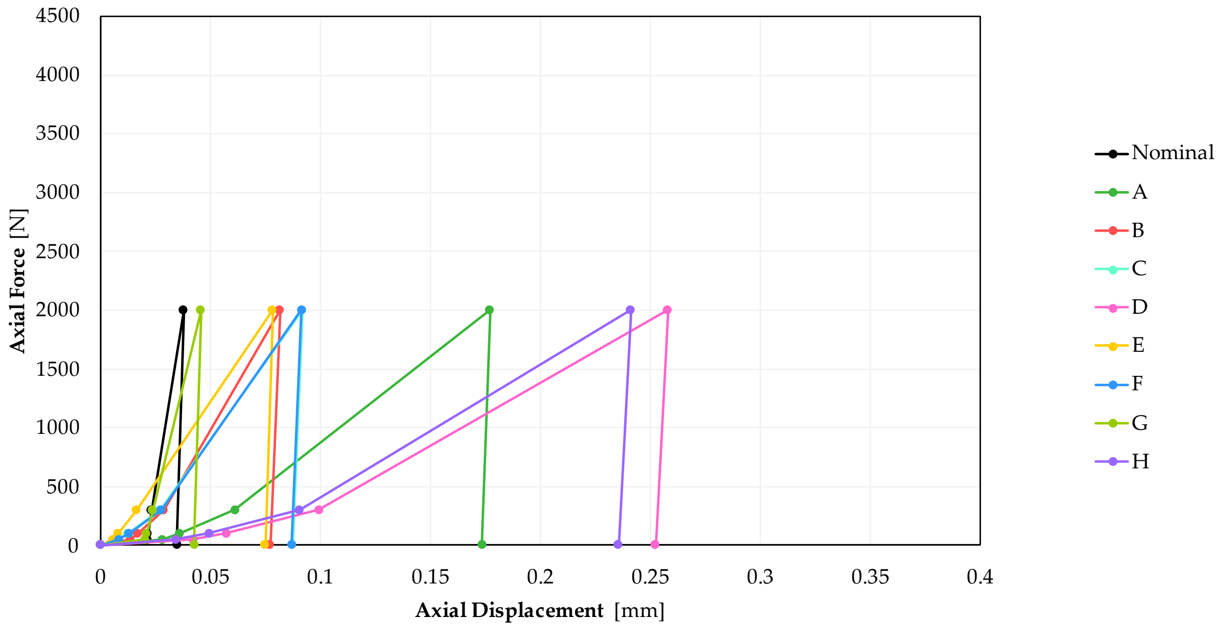

Figure B: Axial Force - Axial Displacement graphs for all models assembled with $F_{ASS} = 2kN$ and then unloaded.

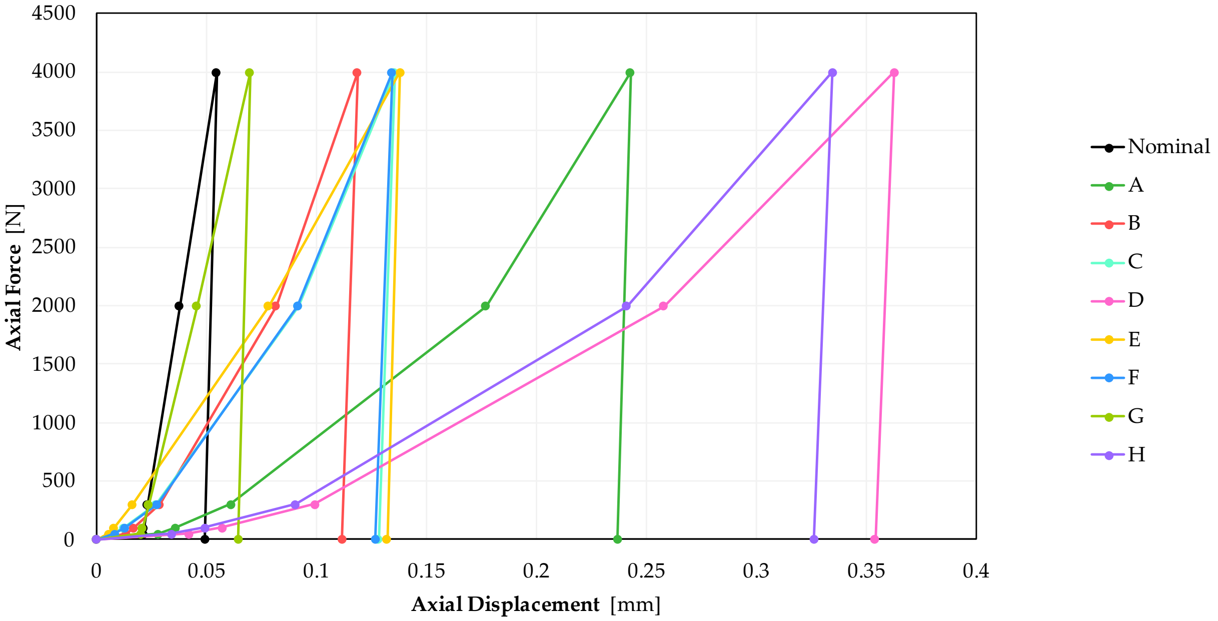

Figure C: Axial Force - Axial Displacement graphs for all models assembled with $F_{ASS} = 4kN$ and then unloaded.

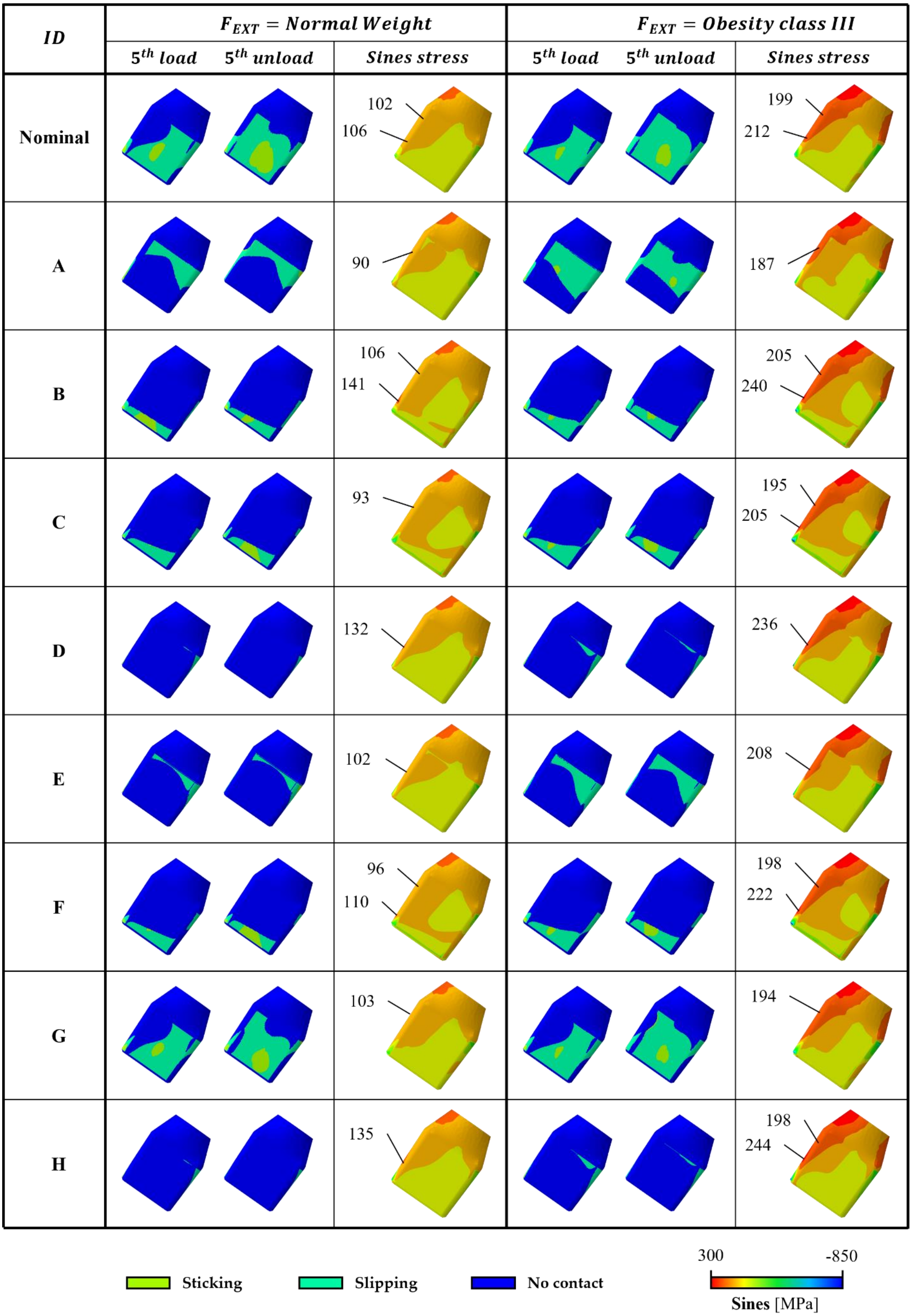

Figure D: CSTATUS and Sines stress color map at the 5th load-unload cycle where lateral maximum values are highlighted. All models are assembled with F_{ASS} = 2kN and then loaded with a normal-weight and a class III obesity respectively on the left and right side. The neck is presented in its anterior (A) view.
